# Key Transitions in the Evolution of Rapid and Slow Growing *Mycobacteria* Identified by Comparative Genomics

**DOI:** 10.3389/fmicb.2019.03019

**Published:** 2020-01-21

**Authors:** Nathan L. Bachmann, Rauf Salamzade, Abigail L. Manson, Richard Whittington, Vitali Sintchenko, Ashlee M. Earl, Ben J. Marais

**Affiliations:** ^1^NSW Mycobacterium Reference Laboratory, Centre for Infectious Diseases and Microbiology Laboratory Services, Institute of Clinical Pathology and Medical Research – Pathology West, Sydney, NSW, Australia; ^2^Centre for Research Excellence in Tuberculosis and the Marie Bashir Institute for Infectious Diseases and Biosecurity, The University of Sydney, Sydney, NSW, Australia; ^3^The Broad Institute of Harvard and MIT, Cambridge, MA, United States; ^4^Sydney School of Veterinary Science, The University of Sydney, Camden, NSW, Australia

**Keywords:** *Mycobacterium* species, growth phenotype, evolution, comparative genomics, phylogenetic analysis

## Abstract

Mycobacteria have been classified into rapid and slow growing phenotypes, but the genetic factors that underlie these growth rate differences are not well understood. We compared the genomes of 157 mycobacterial species, representing all major branches of the mycobacterial phylogenetic tree to identify genes and operons enriched among rapid and slow growing mycobacteria. Overlaying growth phenotype on a phylogenetic tree based on 304 core genes suggested that ancestral mycobacteria had a rapid growth phenotype with a single major evolutionary separation into rapid and slow growing sub-genera. We identified 293 genes enriched among rapid growing sub-genera, including genes encoding for amino acid transport/metabolism (e.g., *livFGMH* operon) and transcription, as well as novel ABC transporters. Loss of the *livFGMH* and ABC transporter operons among slow growing species suggests that reduced cellular amino acid transport may be growth limiting. Comparative genomic analysis suggests that horizontal gene transfer, from non-mycobacterial genera, may have contributed to niche adaptation and pathogenicity, especially among slow growing species. Interestingly, the mammalian cell entry (*mce*) operon was found to be ubiquitous, irrespective of growth phenotype or pathogenicity, although protein sequence homology between rapid and slow growing species was low (<50%). This suggests that the *mce* operon was present in ancestral rapid growing species, but later adapted by slow growing species for use as a mechanism to establish an intra-cellular lifestyle.

## Introduction

Mycobacteria are common environmental organisms, but some species are significant human pathogens. *Mycobacterium tuberculosis* is responsible for tuberculosis in humans (World Health Organization, 2019), and *M. leprae* and *M. lepromatosis*, the cause of leprosy, has become dependent on the human host for survival and dispersal (Monot et al., 2009; Singh et al., 2015). Most mycobacteria are found in soil or water, but they can occupy a variety of environmental niches. Mycobacteria are routinely classified as rapid or slow growers based on their *in vitro* growth characteristics (Kim et al., 2013). Slow growing species typically require more than 7 days before colonies become visible on solid media, while rapid growing species form colonies on selective media within 2–5 days (Kim et al., 2013). The slow growing phenotype has been associated with an intra-cellular lifestyle and pathogenicity, while rapid growing species are mainly environmental and include only a limited number of opportunistic pathogens (Philley and Griffith, 2015). Studies using genetic markers suggested that slow growing species represent a genetically distinct group that evolved from rapid growing species (Wee et al., 2017), while the ancestral *M. abscessus-chelonae* clade is highly divergent from other rapid growing species and have developed unique colonization and disease causing mechanisms (Medjahed et al., 2010; Tortoli, 2012).

The availability of high quality sequences for a large and steadily increasing number of genomes of mycobacterial species in publicly accessible databases provided opportunities for comparative genomics analyses, including a detailed assessment of key differences between rapid and slow growing species. Previous phylogenetic analyses supported a single evolutionary split into rapid and slow growing phenotypes (Tortoli et al., 2017). However, the identification of slow growing species among rapid growers suggested that growth phenotype might be variable and not necessarily related to a major “metabolic transition” (Fedrizzi et al., 2017). In addition, recognition of intermediate growth rates in some phylogenetic clades (e.g., the *M. terrae* complex) demonstrated that growth rate is a complex trait (Vasireddy et al., 2016).

With the goal of providing a better understanding of the evolutionary relationship between the different mycobacterial clades, a new classification scheme has been proposed (Gupta et al., 2018). The proposed scheme redefines all mycobacteria into five sub-genera with members of the distantly related *Abscessus-Chelonae* clade referred to as *Mycobacteroides*, while the majority of other rapid growers are classified as *Mycolicibacterium*. The *M. terrae* complex, which includes slow and intermediate (5–7 days) growers (Tortoli, 2014) were included in the *Mycolicibacter* sub-genus. The vast majority of slow growing species and the major human pathogens, including *M. tuberculosis*, were classified in the *Mycobacterium* sub-genus. However, this analysis did not capture the underlying mechanisms that may explain growth phenotype differences, especially the key differences that occurred during the split between major rapid and slow growth phenotypes.

In order to fill this knowledge gap, we applied pangenome comparative genomics to explore the deep evolutionary origins of mycobacteria, with a specific focus on the genomic differences observed between rapid and slow growing sub-genera.

## Materials and Methods

### Rooting of the Mycobacterial Phylogeny

An initial phylogenetic analysis was performed comparing the five *Mycobacterium* sub-genera against other members of the Actinobacteria phylum, of which the five proposed mycobacteria genera are members. A phylogenetic tree was constructed using 30 conserved genes encoding for ribosomal proteins from the complete genomes of *M. abscessus* (sub-genus *Mycobacteroides*), *M. smegmatis* (sub-genus *Mycolicibacterium), M. sinense* (sub-genus *Mycolicibacter*), *M. triviale* (sub-genus *Mycolicibacillus*) and *M. tuberculosis* (sub-genus *Mycobacterium)*, representing all the major Mycobacteria sub-genera against six Actinobacteria species including *Nocardia brasiliensis* (NZ_KB907307), *Rhodococcus fascians* (NZ_CP015235), *Corynebacterium diphtheriae* (NZ_LN831026) *Amycolatopsis mediterranei* (NC_014318), *Pseudonocardia dioxanivorans* (NC_015312) and *Nakamurella multipartita* (NC_013235.1). *Nakamurella multipartita* was used to root the tree based on previous phylogenetic analysis (Lewin et al., 2016). BLAST and custom scripts were used to extract the nucleotide sequence of 30 conserved genes, which were aligned with Muscle v3.8 (Edgar, 2004) and then concatenated. The concatenated alignment was filtered using Gblocks (Castresana, 2000). The phylogenetic tree was built using the Maximum likelihood method with the General Time Reversible (GTR) model implemented in RaxML (Stamatakis, 2014). Bootstrap values were calculated using 1000 replicates.

### Whole Genome-Based Phylogenetic Analysis

We included all sequenced *Mycobacterium* species with high-quality assemblies available in the NCBI database, as of 31 January 2019. Available genome assemblies were filtered using an assembly quality criteria (<1000 contigs). For each species, the assembly with the smallest number of scaffolds was used as the species representative. The only exception was the use of *M. vulneris* NCXM0100000 instead of the better-assembled *M. vulneris* CCBG00000000, given that the better-assembled genome likely represents a mislabeled species (Tortoli, 2018). Our final dataset included genome assemblies for 157 species (Supplementary Table S1). The genomes were uniformly re-annotated using the Broad Institute‘s prokaryotic annotation pipeline to ensure a consistent annotation protocol for optimal genome comparison.

For detailed assessment of the evolutionary relationship between different *Mycobacterium* species, SynerClust v1 (Georgescu et al., 2018) was employed to perform orthogroup clustering across the 157 assembled sequences, resulting in a set of 304 single-copy core genes. Orthologs were defined as genes that were vertically inherited and have the same function. Sequences for each orthogroup were individually aligned using Muscle v3.8 and then concatenated to build a Maximum likelihood phylogenetic tree using the GTR model with 1000 bootstrap replicates.

### Pangenome Analysis

Since the phylogenetic tree identified a major evolutionary split between rapid and slow growing phenotypes, SynerClust orthogroups (Georgescu et al., 2018) were used to compare the pangenome of rapid versus slow growing mycobacteria on opposite branches of the split. We adopted the proposed sub-genera scheme (Gupta et al., 2018), when referring to different groups of mycobacterial species. Firstly, we used relaxed criteria by searching for orthogroups present in >80% of rapid growing species (sub-genus *Mycolicibacterium*) and in <20% slow growing species (sub-genera *Mycobacterium*, *Mycolicibacillus*, and *Mycolicibacter*). Rapid growing species from the *Mycobacteroides* sub-genus were excluded from this comparative analysis, as they were phylogenetically divergent. Three *Mycolicibacterium* species (*M. doricum, M. farcinogenes*, and *M. tusciae*) with an atypical slow growing phenotype were also excluded. In addition, *M. algericum* from the *Mycolicibacter* sub-genus was excluded as an outlier rapid growing species. However, species with intermediate growth rates in specific environmental conditions were included (Sahraoui et al., 2011). The 80/20 cut-off was used to ensure that genes were not excluded due to poor assembly or other errors and still allowed for accurate identification of genes enriched (uniquely conserved) in rapid growing species (Schreiber et al., 2017). The same approach was used to identify genes enriched among slow growing species.

Uniquely enriched gene clusters, with *q*-values less than 0.05, identified in either rapid or slow growing species, were classified into Clusters of Orthologous Groups (COGs) using WebMGA interface (Wu et al., 2011). COG results were parsed for matches with *e*-value of >1 × 10^–5^ and checked to see if a gene matched to multiple COG models, in which case the model with best match was used. If a COG model was included in multiple classes, then each class was recorded, as it could represent a protein with multiple functional roles. We also confirmed that all members of the gene cluster matched the same COG model. Fisher Exact tests were performed to assess significant differences in the number of functional categories between rapid and slow growing species. Stringent criteria were then applied to search for orthogroups present in both rapid growing sub-genera (*Mycobacteroides* and *Mycolicibacterium*), but absent in all members of the slow growing sub-genera (*Mycolicibacillus*, *Mycolicibacter*, and *Mycobacterium*). The presence and absence of these gene operons across genera was visualized using iTol (Letunic and Bork, 2019).

### Comparative Genomics and Genomic Island Identification

Pairwise whole genome comparison of *M. tuberculosis* H37Rv against selected rapid and slow growing strains was performed using the Artemis comparison tool (Carver et al., 2005) and visualized using the BLAST ring image generator (BRIG) (Alikhan et al., 2011). Two other slow growing (*M. intermedium* and *M. paratuberculosis*) and three rapid growing (*M. smegmatis*, *M. neoaurum*, and *M. fortuitum*) *Mycobacteria* with completed and closed genomes were selected for this analysis to broadly represent both branches of the major evolutionary split that occurred between rapid and slow growing species. *M. tuberculosis* H37Rv was selected as the reference genome for this analysis (Cole et al., 1998; Camus et al., 2002). Regions of differences (RODs) that were identified were compared against the assemblies of the 157 mycobacterial genomes using BLASTn, to observe the distribution across the different sub-genera.

## Results

### Rapid Growing Species Are Ancestral

**FIGURE 1:**
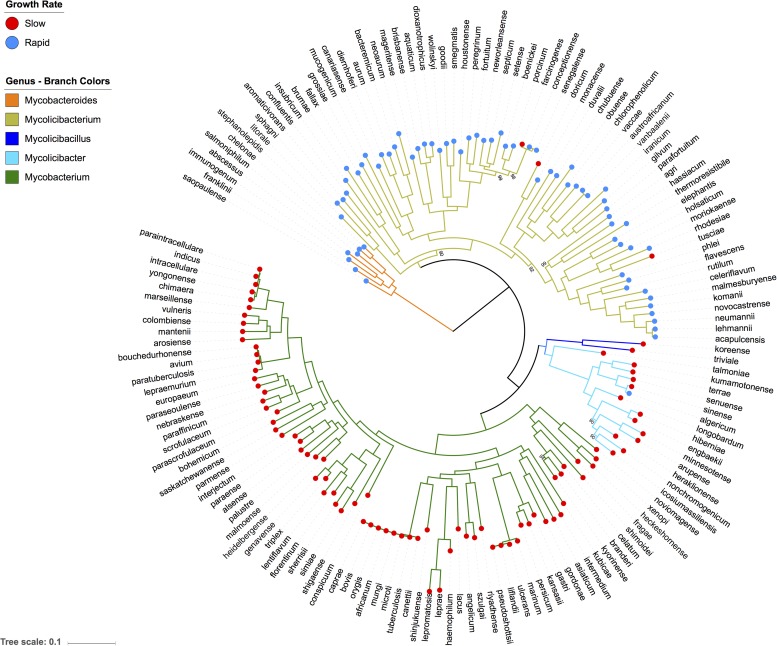
Phylogenetic tree of all well characterised *Mycobacterium* species. This Maximum likelihood tree of 157 well-characterized *Mycobacterium* species is based on nucleotide alignment of 304 single copy genes. It shows five distinct sub-genera and indicates that slow growers evolved from more ancestral fast growing species. Bootstrap values are shown on nodes with less than 100% support.

The phylogeny of Actinobacteria has confirmed that mycobacteria are evolutionary related to *Rhodococcus* and *Nocardia* species (Supplementary Figure S1). It also indicated that the *Mycobacteroides* sub-genus, represented by *M. abscessus* is the most ancestral mycobacteria sub-genus. Therefore, we used the *Mycobacteroides* sub-genus to root our core gene based phylogenetic tree of all 157 mycobacterial genome sequences (Figure 1). Similar to previous findings, our core gene phylogeny identified five major mycobacterial sub-genera (Gupta et al., 2018). Ancestral strains belonging to the *Mycobacteroides* and *Mycolicibacterium* sub-genera included almost all of the rapid growers, with a single evolutionary split separating the vast majority of species with a rapid or slow growing phenotype. The predominantly fast-growing *Mycolicibacterium* sub-genus also contained three slow growing species, *M. doricum*, *M farcinogenes*, and *M. tusciae*, interspersed on separate terminal branches. All other slow growing species were members of the three sub-genera located on the other arm of the major phylogenetic split; *Mycobacterium*, *Mycolicibacillus*, and *Mycolicibacter*. *M. algericum* was the only rapid growing species within these three sub-genera; located on the terminal branch of the *Mycolicibacter* sub-genus.

### Rapid Growing Species Were Enriched in Genes Related to Amino Acid Transport/Metabolism and Transcription

**FIGURE 2:**
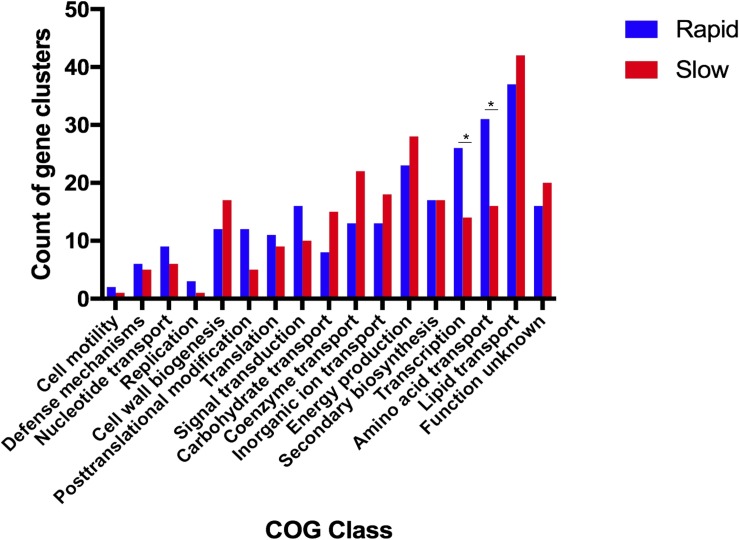
Functions related to amino acid transport and transcription are significantly enriched in the rapid growing mycobacteria. This plot depicts the number of gene clusters that are classified in each Cluster of Orthologous Group (COG) category within both fast-growing (blue) and slow-growing (red) Mycobacteria. ^∗^*P*-value <0.05 as calculated via Fisher Exact Test; COGs classes without ^∗^ are not significantly different between rapid and slow growing species.

We identified 293 genes that were highly enriched among rapid growing species and 309 among slow growing species. Classification of enriched genes into COG functional categories (Figure 2) revealed that genes related to amino acid transport and metabolism (31 genes in rapid growers vs. 16 genes in slow growers) and transcription (26 genes in rapid growers vs. 14 genes in slow growers) were highly enriched among rapid growers.

**FIGURE 3:**
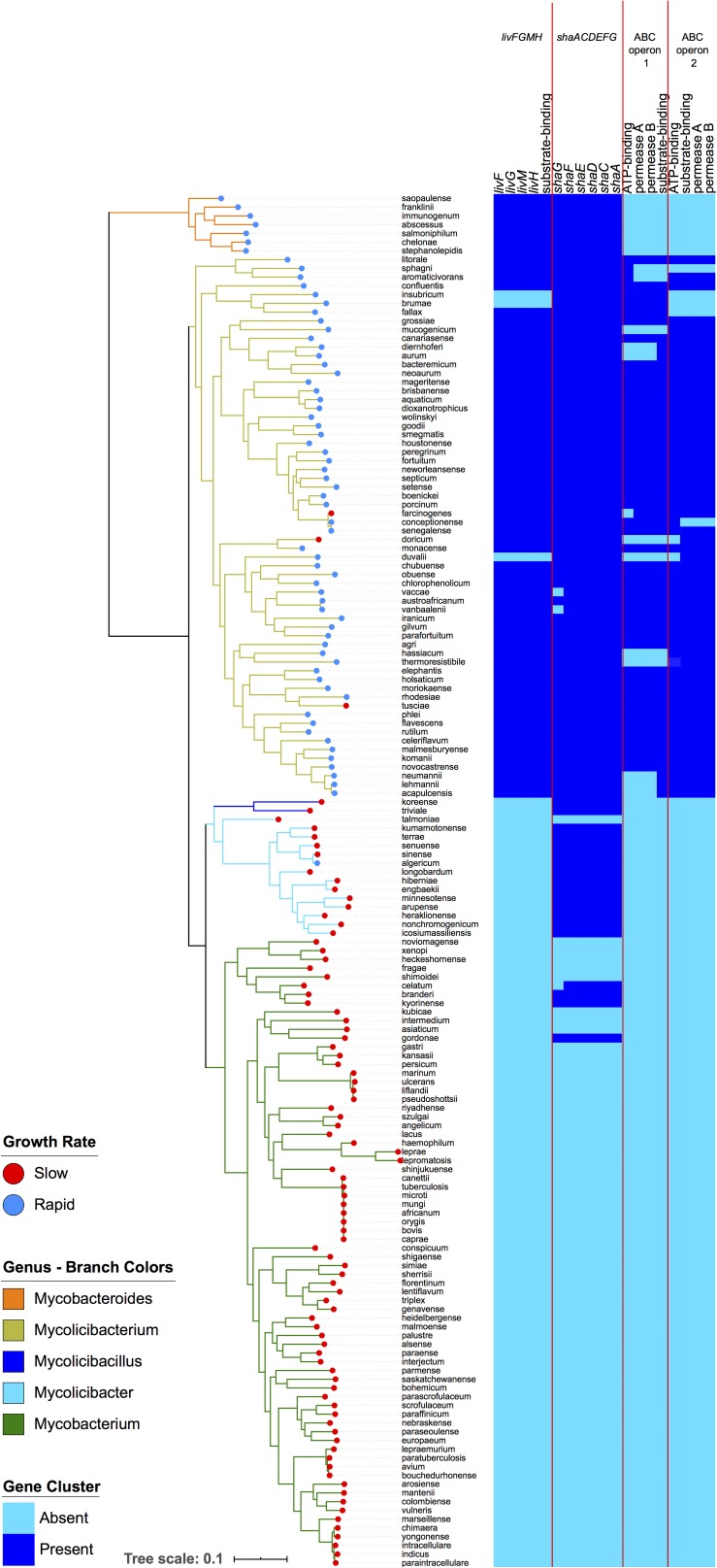
Four operons related to amino acid and ion transport are enriched within rapid growing mycobacteria. The left-hand panel depicts the same phylogeny as in Figure 1. The right panel displays a heatmap showing the presence (dark blue) or absence (light blue) of genes in the four operons. The *livFGMH* operon appears to be missing in *M. insubricum*, *M. brumae*, and *M. duvalii* due to gaps in the assembly.

The enriched and conserved genes in the rapid growing species included 19 genes that are arranged into four operons, all encoding transporter functions (Figure 3). Three of these operons (*livFGMH*, ABC operon 1, and ABC operon 2) were either annotated as amino acid transporters or predicted to be amino acid transporters based on COG annotations (Table 1). The *livFGMH* operon, which consists of five genes that transport branched chain amino acids across the lipid rich mycobacterial cell wall, was universally present in the rapid growing sub-genera *Mycobacteroides* and *Mycolicibacterium*. The other two operons (ABC operon 1 and 2) are uncharacterized ABC transporters predicted by COG annotation to have a role in amino acid transport, and were found in the rapid growing *Mycolicibacterium* sub-genus only. We also noted that the *shaACDEFG* operon, associated with ion transport and pH balance, was uniquely absent from the slow growing *Mycobacterium* genus, which includes most pathogenic species.

**TABLE 1:** Summary of the gene operons enriched in rapid growing species.

	**Gene**		**COG**		
**Operon**	**name**	**Locus tag**	**model**	**Functional annotation**	**COG class**
ABC operon 1	–	MSMEG_6521	COG3842	ABC-type spermidine/putrescine transport systems, ATPase components	Amino acid transport
	–	MSMEG_6522	COG1177	ABC-type spermidine/putrescine transport system, permease component II	Amino acid transport
	–	MSMEG_6523	COG4132	ABC-type uncharacterized transport system, permease component	General function
	–	MSMEG_6524	COG1840	ABC-type Fe3 + transport system, periplasmic component	Inorganic ion transport
ABC operon 2	–	MSMEG_6129	COG2172	Anti-sigma regulatory factor (Ser/Thr protein kinase)	Signal transduction
	–	MSMEG_4354	COG0747	ABC-type dipeptide transport system, periplasmic component	Amino acid transport
	–	MSMEG_4355	COG0601	ABC-type dipeptide/oligopeptide/nickel transport systems, permease components	Amino acid transport
	–	MSMEG_4356	COG1173	ABC-type dipeptide/oligopeptide/nickel transport systems, permease components	Amino acid transport
livFGMH	*livF*	MSMEG_3251	COG0410	ABC-type branched-chain amino acid transport systems, ATPase component	Amino acid transport
	*livG*	MSMEG_3250	COG0411	ABC-type branched-chain amino acid transport systems, ATPase component	Amino acid transport
	*livM*	MSMEG_3249	COG4177	ABC-type branched-chain amino acid transport system, permease component	Amino acid transport
	*livH*	MSMEG_3248	COG0559	Branched-chain amino acid ABC-type transport system, permease components	Amino acid transport
	*livK*	MSMEG_3247	COG0683	ABC-type branched-chain amino acid transport systems, periplasmic component	Amino acid transport
shaACDEFG	*shaG*	MSMEG_0843	COG1320	Multisubunit Na + /H + antiporter, MnhG subunit	Inorganic ion transport
	*shaF*	MSMEG_0844	COG2212	Multisubunit Na + /H + antiporter, MnhF subunit	Inorganic ion transport
	*shaE*	MSMEG_0845	COG1863	Multisubunit Na + /H + antiporter, MnhE subunit	Inorganic ion transport
	*shaD*	MSMEG_0846	COG0651	Formate hydrogenlyase subunit 3/Multisubunit Na + /H + antiporter, MnhD subunit	Inorganic ion transport
	*shaC*	MSMEG_0847	COG1006	Multisubunit Na + /H + antiporter, MnhC subunit	Inorganic ion transport
	*shaA*	MSMEG_0848	COG1009	NADH:ubiquinone oxidoreductase subunit 5, MnhA subunit	Inorganic ion transport
	–	MSMEG_0849	COG0464	ATPases of the AAA + class	Signal transduction

*Locus tags are from Mycolicibacterium smegmatis str. MC2 155 genome (NC_008596).*

Using the stringent criteria, we identified 40 additional gene orthologs present in the majority (>98%) of the members of the *Mycobacteroides* and *Mycolicibacterium* sub-genera, but absent in all members of slow growing species (*Mycobacterium*, *Mycolicibacillus*, and *Mycolicibacter* sub-genera). Unlike the *livFGMH* operon, these genes do not cluster into operons and are scattered across the chromosome. The majority of these genes have functions related to cell respiration (dehydrogenases) and other poorly characterized metabolism roles (Table 2).

**TABLE 2:** Genes conserved in rapid growing sub-genera, but absent in slow growing sub-genera^∗^.

	**Gene**	**COG**	
**Locus tag**	**name**	**model**	**Functional annotation**
MSMEG_0002	Gnd	COG1023	Predicted 6-phosphogluconate dehydrogenase
MSMEG_0096	FabG	COG1028	Dehydrogenases
MSMEG_0248	–	COG0392	Integral membrane protein
MSMEG_0437	yhhQ	COG1738	Uncharacterized member of the PurR regulon
MSMEG_0634	PgpB	COG0671	Membrane-associated phospholipid phosphatase
MSMEG_0829	XthA	COG0708	Exonuclease III
MSMEG_2291	COG4221	COG4221	Short-chain alcohol dehydrogenase
MSMEG_1073	DltE	COG0300	Short-chain dehydrogenases of various substrate specificities
MSMEG_1356	–	No match	Hypothetical protein
MSMEG_1531	COG5516	COG5516	Conserved protein containing a Zn-ribbon-like motif
MSMEG_1540	SrmB	COG0513	Superfamily II DNA and RNA helicases
MSMEG_1656	XthA	COG0708	Exonuclease III
MSMEG_1841	–	COG1773	Rubredoxin
MSMEG_2052	NuoL	COG1009	NADH:ubiquinone oxidoreductase
MSMEG_2511	ViuB	COG2375	Siderophore-interacting protein
MSMEG_3035	Efp	COG0231	Translation elongation factor P
MSMEG_3059	Aes	COG0657	Esterase/lipase
MSMEG_3873	–	COG4178	ABC-type uncharacterized transport system
MSMEG_3873	CobJ	COG1010	Precorrin-3B methylase
MSMEG_4286	LipA	COG0320	Lipoate synthase
MSMEG_4329	–	COG4799	Acetyl-CoA carboxylase, carboxyltransferase component
MSMEG_4342	GloB	COG0491	Zn-dependent hydrolases, including glyoxylases
MSMEG_4484	–	COG1512	Beta-propeller domains of methanol dehydrogenase type
MSMEG_4487	Fur	COG0735	Fe2 + /Zn2 + uptake regulation proteins
MSMEG_4681	–	No match	Hypothetical protein
MSMEG_4898	GutM	COG4578	Glucitol operon activator
MSMEG_4919	–	No match	Hypothetical protein
MSMEG_5007	–	No match	Hypothetical protein
MSMEG_5048	–	No match	Hypothetical protein
MSMEG_5071	–	No match	Hypothetical protein
MSMEG_5083	–	No match	Hypothetical protein
MSMEG_5439	–	COG3583	Hypothetical protein
MSMEG_5443	Ndh	COG1252	NADH dehydrogenase, FAD-containing subunit
MSMEG_5538	PutA	COG1012	NAD-dependent aldehyde dehydrogenases
MSMEG_5720	FadB	COG1250	3-hydroxyacyl-CoA dehydrogenase
MSMEG_5779	PstB	COG1117	ABC-type phosphate transport system, ATPase component
MSMEG_6306	GlnS	COG0008	Glutamyl- and glutaminyl-tRNA synthetases
MSMEG_6313	Tgt	COG0343	Queuine/archaeosine tRNA-ribosyltransferase
MSMEG_6899	–	COG5650	Predicted integral membrane protein
MSMEG_6914	GDB1	COG3408	Glycogen debranching enzyme

*Locus tags are from *Mycolicibacterium smegmatis* str. MC2 155 genome (NC_008596). ^∗^Genes are present in >98% of rapid growing species and 0% in slow growing species.*

To understand additional factors potentially involved in slow growth rate, we sought to identify unique genetic properties shared among the individual terminal branch outliers with a slow-growth phenotype found within the rapid growing *Mycolicibacterium* sub-genus. However, no consistent and significant features could be determined, likely due to the limited number of outlier species with a slow growing phenotype and the possibility of different mechanisms employed by each outlier, given that they are each located on a terminal branch.

### Mammalian Cell Entry (*mce1*) Operon Found in Rapid and Slow Growing Mycobacteria

**FIGURE 4:**
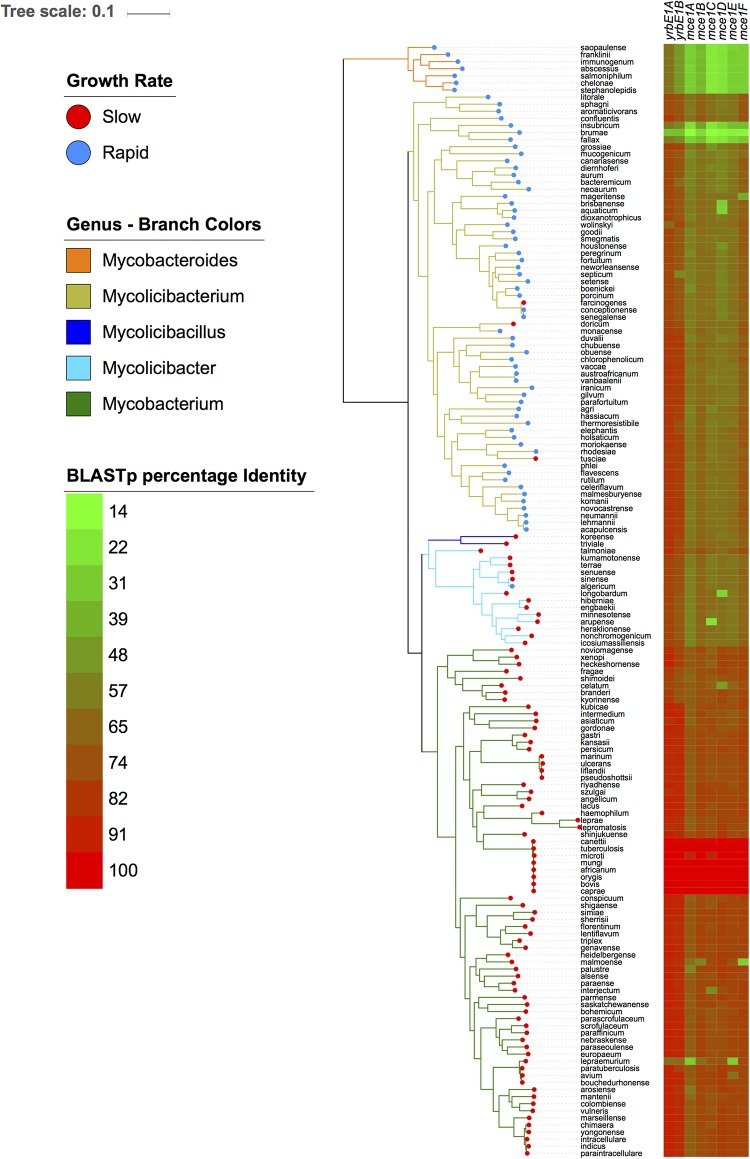
The *mce1* operon is highly variable across all five mycobacterial sub-genera. The left-hand panel depicts the phylogeny as in Figure 1. The right panel shows a gradient heatmap based on protein sequence identity for each protein encoded by the *mce1* operon using BLASTp.

Among the 309 enriched genes in the slow growing species, genes associated with the ESX-5 Type VII secretion system, PPE family and the mammalian cell entry (*mce*) operon were most prevalent. The *mce1* operon is comprised of two *yrbE* (*yrbE1A* and *yrbE1B*) and six *mce* genes (*mceA1, mceB1, mceC1, mceE1*, and *mceF1*) (Zhang and Xie, 2011). Homologs of the *mce1* operon were also detected in rapid growing species, although the gene clustering approach used in this study separated the rapid grower homologs into a single ortholog group. In order to examine the differences in the *mce1* operon in more detail, the protein sequences encoded by the *mce1* genes were extracted from the genome of *M. tuberculosis* H37Rv and BLASTp was used to assess the sequence variability across the all 157 genomes (Figure 4). Within the *Mycobacterium* sub-genus, *mce1* proteins shared >80% sequence identity, with almost 100% identity among species belonging to the *M. tuberculosis* complex. Shared sequence identity with the *Mycolicibacillus*, *Mycolicibacter*, and *Mycolicibacterium* sub-genera was significantly reduced for the six *mce* genes (<60%). The two *yrbE* genes, which encodes for membrane proteins similar to ABC transporter permeases, have homologs with sequence identity in the range of 65–75% in most *Mycobacterium* species; however, sequence homology with the ancestral *Mycobacteroides* sub-genus was less than 14%.

### Horizontal Gene Transfer in Slow Growing Mycobacteria

**FIGURE 5:**
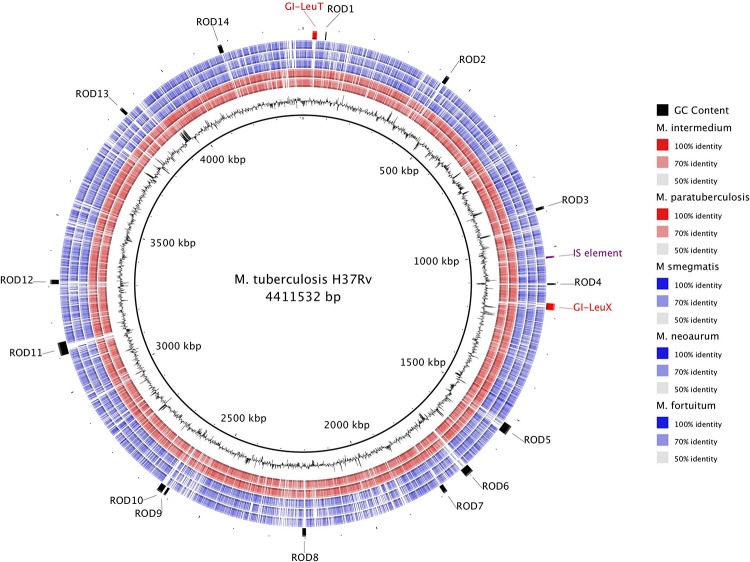
Genomic comparison of selected slow and rapid growing mycobacteria highlights the role of horizontal gene transfer in the evolution of slow growers. The innermost ring depicts the *M. tuberculosis* H37Rv genome coordinates in kilobases (kbp). The next ring shows the GC Content (black). The remaining rings show BLASTn comparison with H37Rv against five other mycobacterial assemblies (from inner to outer: *M. intermedium*, *M. paratuberculosis*, *M. smegmatis*, *M. neoaurum*, and *M. fortuitum;* see section “Materials and Methods”). BLAST matches with nucleotide identity 50–100% are colored, while non-matching regions are white. The outer ring contains annotation of specific regions of difference (ROD) found in *M. tuberculosis* that likely represent acquisition by horizontal gene transfer.

It has been hypothesized that horizontal gene transfer (HGT) played an important role in the evolution of pathogenic slow growing phenotypes. In order to investigate the role of HGT in the evolution of slow growing species, we performed a whole-genome alignment using BLAST of the well-annotated *M. tuberculosis* H37Rv genome compared against two other slow growing pathogens (*M. intermedium* and *M. paratuberculosis)* and three rapid growing species (*M. smegmatis*, *M. neoaurum*, and *M. fortuitum)* to identify regions where elements have inserted into the *M. tuberculosis* H37Rv genome (Figure 5). In total, 16 RODs were identified and BLASTn was used to confirm the distribution of these RODs in the other 157 genomes.

Six RODs (GI-LeuT, ROD-1, ROD-2, ROD-5, ROD-6, and ROD-10) are present in at least 70% of slow growing species, with notable absences in *M. leprae* and *M. lepromatosis*, which have undergone significant genome reduction (Monot et al., 2009; Singh et al., 2015). Matches to these regions were observed in <10% of rapid growing species. The other 10 RODs were either found to be exclusive to *M. tuberculosis* complex, which is comprised of a group of almost clonal species including *M. bovis* and *M. africanum*, or sporadic in both slow and rapid growing genomes (Supplementary Table S2).

One of the RODs commonly found in slow growers, but missing from rapid growers, was identified as a potential genomic island (GI-LeuT) containing genes that encode for biofilm regulators and a number of hypothetical proteins. The other five RODs contained genes encoding hypothetical proteins ROD-1 and ROD-6 and/or PPE family proteins (ROD-2 and ROD-5), while ROD-10 encodes a number of membrane proteins (PPE and ESX secretion proteins) involved in host-pathogen interactions. A second genomic island GI-LeuX, found only in species of the *M. tuberculosis* complex, carried three transposase genes along with ESX and PPE genes. Other RODs found only in the *M. tuberculosis* complex include ROD-4, which encodes for an ABC transporter and is associated with a spike in the GC content indicating likely acquisition via HGT. PPE family proteins were encoded on ROD-9, ROD-13, and ROD-14. ROD-11 encodes a single toxin-antitoxin system and contains a Clustered Regularly Interspaced Short Palindromic Repeat (CRISPR) region (Freidlin et al., 2017). ROD-8 is located near the *mce1* operon and encodes multiple toxin-antitoxin systems.

## Discussion

This report illustrates the capacity of comparative genomics to explain differences between mycobacterial growth phenotypes using comprehensive sets of high-quality genomes. The phylogenetic tree presented here was built from a concatenated alignment of 304 single copy genes from ortholog clusters generated using a novel Synerclust-based orthogroup method, which allowed us to leverage genomic synteny to help identify orthologs across species. This high resolution phylogenetic tree was congruent with previous phylogenetic analyses that used different methods and separated mycobacteria into five major sub-genera (Fedrizzi et al., 2017; Tortoli et al., 2017; Gupta et al., 2018). The novel comparative genomics methods employed allowed us to focus with high granularity on key genetic differences between rapid and slow growing species.

Our detailed mycobacterial phylogeny suggests a single deep evolutionary split between rapid and slow growing species, with ancestral species being rapid growers, with only a handful of terminal branch exceptions. These include *M. farcinogenes*, *M. doricum*, and *M. tusciae* being slow growing species located within a rapid sub-genus and *M. algericum* identified as a rapid growing *M. terrae*-complex species located within the slow and intermediate growing *Mycolicibacter* sub-genus (Sahraoui et al., 2011). *M. tusciae* and *M. doricum* both exhibit substantially slower growth rates than other members of their fast-growing clade. *M. tusciae* takes 4 weeks to grow on Middlebrook agar (Tortoli et al., 1999), and *M. doricum* takes 2 weeks on Lowenstein-Jensen medium (Tortoli et al., 2001). This suggested ongoing growth phenotype evolution, which is also illustrated by *M. farcinogenes* that forms colonies in 5–10 days compared to the closely related *M. senegalense* that forms colonies in 2–5 days (Hamid, 2014). However, switches in growth phenotypes are rare and the majority of species within specific sub-genera maintain the growth rate of their ancestors, with a single major evolutionary transition from rapid to slow growing phenotypes.

This study focussed on genomic differences between the rapid growing *Mycolicibacterium* sub-genus and slow growing sub-genera on the other branch of this evolutionary split, excluding species with a disco crdant growth phenotype. It was found that amino acid transporters were highly conserved in rapid growing species, but absent in slow growing species. Amino acid transporters are membrane-bound proteins that mediate the transfer of amino acids into and out of cells, with critical roles in regulating energy metabolism, protein synthesis and redox balance (Kandasamy et al., 2018). The ATP-binding *livFGMH* operon specifically transports branched chain amino acids such as leucine, isoleucine and valine, which are important for bacterial growth (Conner and Hansen, 1967). The *livFGMH* operon is conserved in all rapid growing species, including the outgroup *Mycobacteroides* sub-genus, but absent from all slow growing sub-genera. Using stricter cut-offs the enriched orthogroups were filtered to identify an additional 40 genes found only in rapid growing species (Table 2). The majority of these genes have predicted dehydrogenase functions, although their metabolic functions remains poorly characterized.

Importantly, this study identified two ATP-binding transporter operons unique to rapid growers, predicted to be involved in amino acid transport based on COG annotations. Both operons consisted of four genes, including an ATP-binding protein, substrate binding protein and two permeases (transmembrane proteins). ABC transporters are amongst the most common ATP powered transporters found in bacteria and are linked to the transport of a wide variety of substrates and lipids across the membrane bilayer (Ford and Beis, 2019). The ability to utilize a variety of substrates and metabolites are likely to be important for sustaining rapid growth (Kandasamy et al., 2018), with a strong signal that sub-optimal amino acid transport may be particularly growth limiting. These two ABC operons were found to be conserved only in the *Mycolicibacterium sub-*genus and absent in *Mycobacteroides*. They were seemingly acquired when mycobacteria diverged from the *Mycobacteroides* outgroup and were then lost from species that evolved into slow growers. The likely gain and loss of these transporters, during this key evolutionary transition, increases interest in their specific function.

The *shaACDEFG* operon was selectively absent from the more pathogenic *Mycobacterium* sub-genus. It encodes a Na + /H + antiporter that regulates cellular pH under extreme conditions (Kosono et al., 2005). A recently published genomic comparison of 28 rapid and slow growing species revealed several genes making up the *livFGMH* and *shaACDEFG* operons, and genes that encode Msp porins, to be absent in pathogenic *Mycobacterium* species (Wee et al., 2017). However, this small study excluded other slow growing genera and our study demonstrates that, unlike the *livFGMH* operon that is universally absent from all slow growing genera, the *shaACDEFG* operon is only absent from the more pathogenic *Mycobacterium* sub-genus. This would suggest that the *shaACDEFG* operon was deleted as mycobacteria developed a more pathogenic intracellular lifestyle.

A key virulence mechanism for *M. tuberculosis* is the ability to invade host macrophages, which is in part mediated by the mammalian cell entry (*mce1)* operon. While the *mce1* operon is considered a key feature of slow growing pathogenic mycobacteria, homologs of the *yrbE* and *mce* genes are also found in distant rapid growing species such as *M. smegmatis* and *M. abscessus* (Kumar et al., 2005; Sassi and Drancourt, 2014). Interestingly, the gene clustering approach used in this study divided the *mce1* operon from rapid and slow growing species into two ortholog clusters, with limited homology between the slow growing *M. tuberculosis* complex and the ancestral rapid growing *M. abscessus-chelonae* complex. This level of variability suggests that the *mce1* operon probably fulfilled a different function in ancestral rapid growing species. It may have provided ancestral mycobacteria with the ability to enter amoeba cells (Zhang and Xie, 2011), paving the way for the adoption of a pathogenic intra-cellular lifestyle with ongoing evolution of the *mce1* operon facilitating macrophage entry. The potential advantage afforded is supported by the presence of four duplicated *mce1* operons in pathogenic mycobacterial species, which suggests strong positive selective pressure. It has been suggested that the smaller genome size observed in more pathogenic slow growers resulted from the loss of genes required for essential nutrient uptake and metabolism in the environment (Devulder et al., 2005), which were no longer required after they acquired the ability to live within host cells.

In contrast to our observation of whole operons uniquely conserved in rapid growers, we identified only scattered genes coding for the ESX and PPE protein families to be highly enriched among slow growers. These genes produce proteins that are involved in host interaction and are mainly secreted by ESX Type VII secretion systems, which are considered to be important for virulence (Simeone et al., 2015). Numerous hypothetical and metabolic genes were also detected on 16 genomic islands that likely represent horizontal gene transfer; six were common in slow growing species but rarely observed in rapid growers. Evidence of horizontal gene transfer includes the presence of a tRNA at the boundary of the island, as well as the presence of genes encoding transposase and distinct differences in GC content.

Several of the islands identified are consistent with islands found using an automated detection method for the *M. tuberculosis* genome (Becq et al., 2007), but we detected several additional genomic islands (ROD-1, 2, 5, 6, 7, 12). In particular, ROD-1 carries genes that encode biofilm regulators and an ESX secretion protein that is an ESX Type VII secretion system substrate. The ESX Type VII secretion system has been shown to be critical for virulence (Houben et al., 2012, 2014). ROD-2, which encodes highly polymorphic PPE proteins, offers another example of horizontal gene transfer in slow growers. The ROD comparison was limited by the inclusion of only select high quality assembled genomes, since fragmented genomes will lower the accuracy of genomic islands detection. Although the RODs identified would be influenced by strain selection, strains were selected from representative metabolic groups and divergent branches of the evolutionary tree to maximize the insight gained.

## Conclusion

In conclusion, this study provides a detailed description of key genetic differences using a novel comparative genomics approach. We identified four operons that are uniquely conserved in rapid growing sub-genera of Mycobacteria, which could be targeted in future microbiological and pharmacological studies to elucidate their role in growth phenotype determination. Potential genes that could be knocked out or silenced to observe growth rate differences include those linked to amino acid transport, such as the *livFGMH* and ABC transporter operons. The *mce-1* operon was found to be ubiquitous among mycobacteria, but significantly evolved among slow growing species with ability for sustained intra-cellular survival, suggesting a crucial role in this lifestyle transition.

## Data Availability Statement

Publicly available datasets were analyzed in this study. This data can be found here: on https://www.ncbi.nlm.nih.gov/ with accession numbers: NC_010397.1, NZ_CP007220.1, NZ_MAFQ00000000.1, NZ_CP011530.1, GCA_002013645.1, NZ_CP010271.1, NZ_AP018165.1, NZ_FLMU00000000.1, NZ_PDCP00000000.1, NZ_MVHF00000000.1, NZ_JALN 00000000.2, NZ_LT549889.1, NZ_CCAW000000000.1, NZ_ MVHJ00000000.1, GCA_002553535.1, NZ_BCSX00000000.1, NZ_FJNX00000000.1, NZ_LQOL00000000.1, NZ_MVHN00000000.1, NZ_JYNL00000000.1, NC_018027.1, GCA_001077745.1, NZ_LQOQ00000000.1, GCA_001907655.1, NZ_CP020809.1, NZ_LQOS00000000.1, NZ_PDCQ00000000.1, NZ_ LBNO00000000.1, NZ_LQOJ00000000.1, NZ_CCAY000000000.1, NZ_MIHA00000000.1, NZ_CP011269.1, NC_014814.1, NZ_CP012150.1, NZ_MCHX00000000.1, NZ_ARBU00000000.1, NZ_MIGZ00000000.1, NZ_FJVO00000000.1, NZ_MV HS00000000.1, NZ_LQPC00000000.1, NZ_CVTA00000000.1, NZ_NKCN00000000.1, NZ_CP019882, NZ_CCBF000000000.1, NZ_CVTB00000000.1, NZ_MVIA00000000.1, NZ_MVIB00000000.1, NZ_CYSI00000000.1, NC_023036.2, NZ_NKCO00000000.1, NZ_CWKH00000000.1, NZ_BCTA00000000.1, NZ_JYN U00000000.1, NZ_MVID00000000.1, GCA_001403655.1, NZ_C P014475.1, GCA_001942045.1, NC_016604.1, GCA_900108565.1, GCA_001021425.1, NZ_CBMO000000000.1, GCA_900236745.1, NC_008596.1, NZ_NOZR00000000.1, NZ_AGVE00000000.1, NZ_MVIM00000000.1, NZ_CP011491.1, NC_008726.1, NZ_LQQA00000000.1, NZ_NCXO00000000.1, GCA_002102395.1, NZ_MVHC00000000.1, NZ_LASW00000000.2, NZ_LQOT 00000000.1, NZ_LDPO00000000.1, NZ_LQOZ00000000.1, NZ_ FJVP00000000.1, GCA_001679965.1, NZ_LQPG00000000.1, NZ_MVHZ00000000.1, GCA_002101775.1, NZ_LQPS00000000.1, NC_015576.1, NZ_MLQM00000000.1, NZ_LT906469.1, NC_015758.1, NZ_MVHD00000000.1, NZ_MVHE00000000.1, NZ_MVHG00000000.1, NZ_CCBD000000000.1, NC_008595, GCA_001053185.1, NZ_MVHL00000000.1, NC_002945.4, NZ_ MVHM00000000.1, NC_015848.1, NZ_CP016401.1, GCA_000974705.1, NZ_CP012885.2, NZ_CP020821.1, NZ_LQOR00000000.1, NZ_CTEC00000000.1, NZ_LQOV00000000.1, NZ_LQO W00000000.1, GCA_002102175.1, NZ_JAGZ00000000.1, NZ_ LQOY00000000.1, NZ_CP011883.2, GCA_001077755.1, NZ_ MVHR00000000.1, NC_018612.1, NZ_FJVQ00000000.1, GCA_ 001722345.1, NC_016946.1, NC_022663.1, NZ_LQPD00000000.1, GCA_000759695.1, NZ_LQPF00000000.1, NZ_CTEE00000000.1, NC_002677.1, CP021238.1, GCA_000966355.1, NC_020133.1, NZ_MVHV00000000.1, NZ_MVHW00000000.1, NZ_ HG917972.2, NZ_CP023147.1, CP010333.1, NZ_LXTB00000000.1, GCA_001021495.1, NZ_MVIC00000000.1, NZ_APKD00000000.1, NZ_LQPJ00000000.1, GCA_002101815.1, NZ_MPNT 00000000.1, NC_016948.1, NZ_ADNV00000000.1, NZ_MVIE 00000000.1, NC_002944.2, NZ_LQPO00000000.1, NZ_MVIF 00000000.1, NZ_BCND00000000.1, NZ_LQPQ00000000.1, NZ_ LQPR00000000.1, GCA_001667885.1, GCA_001722325.1, NZ_ AP018164.1, NZ_LQPU00000000.1, NZ_MVIK00000000.1, NZ_CP010996.1, NZ_LQPW00000000.1, NZ_CCAU000000000.1, NC_000962.3, NC_008611.1, NCXM00000000.1, GCA_ 002102015.1, and NC_021715.1.

## Author Contributions

NB, BM, VS, RW, AM, and AE developed the research aims and the concept for this manuscript. NB, RS, and AM performed the analysis. NB and RS generated the figures and tables. NB, BM, AE, AM, VS, and RW provided the interpretation of results. NB, BM, and AM wrote the manuscript. All authors read and approved the final version of the manuscript.

## Conflict of Interest

The authors declare that the research was conducted in the absence of any commercial or financial relationships that could be construed as a potential conflict of interest.
