# Supplementary figures and images for: Key Transitions in the Evolution of Rapid and Slow Growing Mycobacteria Identified by Comparative Genomics
Source: Front Microbiol. 2020 Jan 21;10:3019. doi: 10.3389/fmicb.2019.03019 (PMC6985099; doi:10.3389/fmicb.2019.03019)

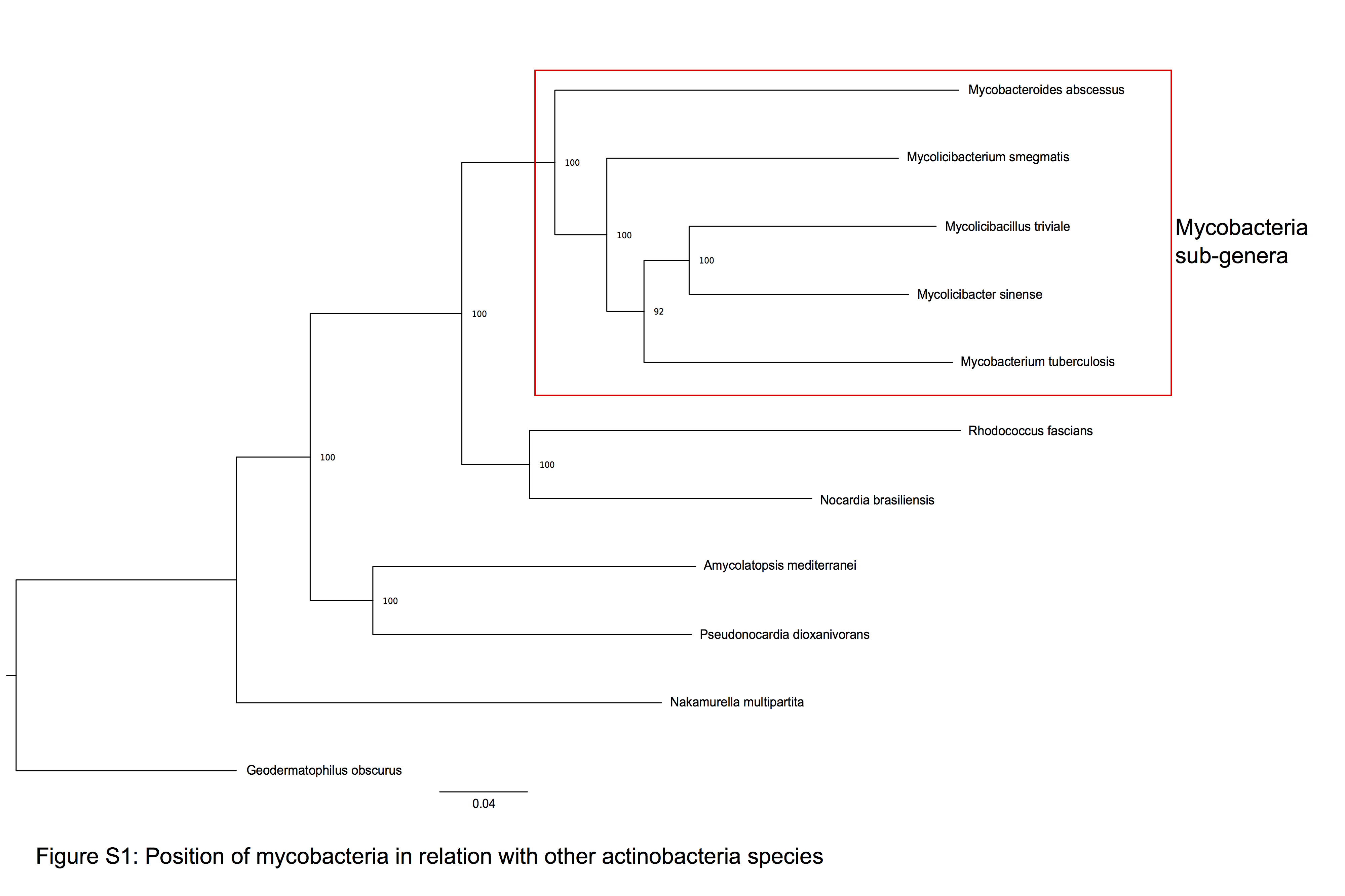

Supplement: Supplementary file 1 [file Image_1.TIFF]
